# Caveolin-1 Variant Is Associated With the Metabolic Syndrome in Kuwaiti Children

**DOI:** 10.3389/fgene.2018.00689

**Published:** 2018-12-21

**Authors:** Rasheeba Nizam, Ebaa Al-Ozairi, Jo Max Goodson, Motesam Melhem, Lena Davidsson, Hessa Alkhandari, Ashraf Al Madhoun, Sara Shamsah, Malak Qaddoumi, Ghazi Alghanim, Nouf Alhasawi, Mohamed Abu-Farha, Jehad Abubaker, Ping Shi, Mor-Li Hartman, Mary Tavares, Milad Bitar, Hamad Ali, Hossein Arefanian, Sriraman Devarajan, Faisal Al-Refaei, Osama Alsmadi, Jaakko Tuomilehto, Fahd Al-Mulla

**Affiliations:** ^1^Functional Genomics Unit, Dasman Diabetes Institute, Kuwait City, Kuwait; ^2^Clinical Division, Dasman Diabetes Institute, Kuwait City, Kuwait; ^3^Applied Oral Sciences, The Forsyth Institute, Cambridge, MA, United States; ^4^Family Medicine and Pediatric Unit, Dasman Diabetes Institute, Kuwait City, Kuwait; ^5^Faculty of Allied Health Sciences, Kuwait University, Kuwait City, Kuwait; ^6^Biochemistry and Molecular Biology Unit, Dasman Diabetes Institute, Kuwait City, Kuwait; ^7^Faculty of Medicine, Kuwait University, Kuwait City, Kuwait; ^8^Immunology Unit, Dasman Diabetes Institute, Kuwait City, Kuwait; ^9^National Dasman Diabetes Biobank, Dasman Diabetes Institute, Kuwait City, Kuwait; ^10^Cell Therapy and Applied Genomics, King Hussein Cancer Center, Amman, Jordan; ^11^Research Division, Dasman Diabetes Institute, Kuwait City, Kuwait

**Keywords:** *CAV1*, HDLC, metabolic syndrome, Kuwaiti children, obesity

## Abstract

Caveolin-1 (*CAV1*) variants have been suggested to be associated with obesity and related metabolic disorders, but information based on human studies is limited. In the present study, we aimed to investigate the potential association between the *CAV1* rs1997623 C/A variant and metabolic syndrome (MetS) in Kuwaiti children. DNA from saliva samples collected from 1313 Kuwaiti children (mean age: 12 years) were genotyped using the TaqMan SNP genotyping assay. The classification of MetS was based on the presence/absence of four indicators; (1) central obesity, (2) elevated systolic or diastolic blood pressure, (3) low salivary high-density lipoprotein cholesterol (HDLC), and (4) high salivary glucose. In this study, children with MetS scored ≥3, children in the intermediate metabolic group scored 1 or 2 and children without MetS scored 0. About one-third of the children were obese. A total of 246 children (18.7%) were classified as having MetS; 834 children (63.5%) were in the intermediate metabolic group, and 233 children (17.7%) had no indication of MetS. Obesity was highly prevalent in the MetS group (91.9%) while 26.8% of children were obese in the intermediate metabolic group. None of the children were obese in the group without MetS. Analysis of the *CAV1* rs1997623 variant revealed a significant association of the A-allele (*p* = 0.01, Odds Ratio (OR) = 1.66) and the heterozygous CA-genotype (*p* = 0.005, OR = 1.88) with MetS. Consistently, the A-allele (*p* = 0.002, OR = 1.71) and CA-genotype (*p* = 0.005, OR = 1.70) also showed significant association with the intermediate metabolic group. Furthermore, the A-allele (*p* = 0.01, OR = 1.33) and the CA-genotype (*p* = 0.008, OR = 1.55) were associated with low levels of saliva HDLC. Individuals who were heterozygous or homozygous for the variant (CA/AA) showed significantly lower levels of high HDLC compared to those harboring the CC-genotype (*p* = 0.023). Our study revealed a novel association of the *CAV1* rs1997623 variant with the MetS and with low saliva HDLC levels in young Kuwaiti children and indicated the need for further in-depth studies to unravel the role of *CAV1* gene in the genetic etiology of MetS.

## Introduction

The prevalence of obesity has dramatically increased over the past decades, affecting nearly half of the Kuwaiti population (ALNohair, 2014). Childhood obesity is considered one of the major public health concerns in many countries including Kuwait due to its long-term effects on health and wellbeing of the individuals in the population. Recent reports on Kuwait Nutrition Surveillance System indicates that 26.2% of school children (>5–19 years old) are obese according to the WHO Body Mass Index (BMI) for age *z*-scores, indicating a high prevalence of childhood obesity and its associated metabolic complications (MOH, 2015). Metabolic syndrome (MetS) refers to a cluster of interconnected factors that increase the risk of non-communicable diseases, typically defined based on clinical measurements such as blood pressure and abdominal obesity as well as fasting blood glucose, serum triglycerides, and high-density cholesterol concentrations. Very limited information is currently available on the metabolic health status of children in Kuwait (Elkum et al., 2016).

The etiology of obesity is multifactorial and includes both genetic and environmental factors. Global studies have reported 157 diverse genetic loci and other lifestyle-related susceptibility factors (Willer et al., 2013). In this post-genome-wide association study era, an enormous wealth of genomic data has become publicly available, enabling the fine-mapping and translation of genetic information into clinical practice. In this study, we focused on the caveolin-1 (*CAV1*) gene, located on chromosome 7q31.2, that acts as an integral component of the small plasma membrane invaginations known as caveolae (Nixon et al., 2007). Caveolae are implicated in endocytosis and in other signaling pathways involving eNOS, G Beta Gamma, G alpha, integrin, platelet-derived growth factor, and endocytic virus entry (Schwencke et al., 2006). Several animal cell model studies have elucidated the role of *CAV1* in glucose and lipid metabolism. *CAV1* tends to play an important role in blood glucose regulation either by directly interacting with the insulin receptor or by facilitating *GLUT4*-mediated glucose transport (Gonzalez-Munoz et al., 2009). Upregulation of *CAV1* has been linked to a heightened state of oxidative stress and impaired diabetic wound healing (Bitar et al., 2013). *CAV1*-deficient mice have been reported to exhibit increased levels of triglycerides and free fatty acids, predisposing them to hyperlipidaemia and obesity (Park et al., 2002). Conflicting roles of *CAV1* in promoting or negatively regulating HDLC uptake have also been reported in literature (Langmann et al., 1999; Matveev et al., 1999; Frank et al., 2002; Wang et al., 2003).

Caveolin-1 variants have been previously implicated in various cancers, such as breast, renal and nasopharyngeal carcinomas (Liu et al., 2011; Tsou et al., 2011; Chang et al., 2014). Nevertheless, very few studies have explored the significance of the association of *CAV1* variants with metabolic traits (Pojoga et al., 2011; Baudrand et al., 2015; Chen et al., 2016). The *CAV1* rs926198 SNP was reported to be associated with MetS in Caucasians and Hispanics (Baudrand et al., 2015). The rs926198 and rs3807989 SNPs were also shown to be associated with insulin resistance and hypertension in the same study population (Pojoga et al., 2011; Baudrand et al., 2015). Similarly, the rs3807989 SNP was reported to be associated with significant risk of coronary heart disease in the Chinese Han population (Chen et al., 2016). Nevertheless, no study has so far characterized the role of *CAV1* rs1997623 variant with MetS.

## Materials and Methods

### Study Rationale

A review of the literature indicates several animal and cell model studies depicting the significance of CAV1 in the pathophysiology of diabetes and obesity. Our previous study has additionally shown the risk role of CAV1 in impaired diabetic wound healing (Bitar et al., 2013). In preparation for the present study, we shortlisted CAV1 rs1997623 single nucleotide polymorphism as a population-specific risk variant from our in-house exome database consisting of 156 adult Arabs. We were intrigued to note that the current genome wide association (GWA) studies did not highlight any role for CAV1 in MetS. A detailed search indicated that the most commonly used Illumina Human OmniExpress Bead Chip array does not include the tested rs1997623 variant. Therefore, we adopted a candidate gene approach to evaluate the risk association of the CAV1 rs1997623 SNP with indicators of metabolic complications associated with childhood obesity. Saliva samples were used as specimens to analyze the association of the candidate variant with metabolic complications related to obesity. Parallel validation studies have been reported in the literature indicating the possibility of saliva to be a non-invasive surrogate marker for blood glucose and blood lipid measures (Mascarenhas et al., 2014; Singh et al., 2014; Shi et al., 2015; Hartman et al., 2016). The study population consisted of a sub-sample of Kuwaiti children drawn from previous studies (Goodson et al., 2013, 2014; Shi et al., 2015; Hartman et al., 2016) and used information collected during the original study. The risk variant was investigated for its association with MetS based on indicators such as waist circumference, blood pressure (diastolic and systolic), saliva glucose concentration and saliva HDLC concentration.

### Sample Collection

A cohort of Kuwaiti school children were recruited for the nationwide “Kuwait healthy lifestyle study” from all six Kuwaiti governorates covering 138 schools from 2011 to 2014 (Goodson et al., 2013, 2014, 2017; Shi et al., 2015; Hartman et al., 2016). A total of 8317 children were enrolled into the original study. The average number of schools visited was 23 schools per governorate (range 13–31, average of 46 children/school). For the present study, a subset of 1313 participant were chosen by random sampling. Written informed consent was obtained from the guardians/parents of all participants in the original study in accordance with the Declaration of Helsinki. Participant’s assent was obtained on the day of the visit. The original study as well as the present study were reviewed and approved by the institutional research ethics committees at Dasman Diabetes Institute.

Each participant provided a sample of saliva (3 mL) after an overnight fast and biochemical analyses were performed as described previously (Goodson et al., 2013, 2014, 2017; Shi et al., 2015; Hartman et al., 2016). DNA was extracted using the QIAamp^®^ DNA extraction kit from Qiagen (Hilden, Germany) according to the manufacturer’s instructions. Previously recorded anthropometric, clinical and biochemical parameters included body height (m) and weight (kg), BMI (kg/m^2^), heart rate (HR, beats/min), self-reported hours of sleep, saliva flow rate (SFR, mL/h), waist circumference (WC, cm), systolic blood pressure (SBP, mmHg), diastolic blood pressure (DBP, mmHg), saliva glucose concentration (mg/dL), and saliva HDLC concentration (mg/dL) are presented in (Table 1). Study participants fitness was assessed based on the degree of HR elevation following 3 min of standardized exercise. All methods and cut-offs are presented by Shi et al. (2015). Obesity was defined as BMI for age *z*-scores ≥95th percentile according to the WHO. Central obesity was based on WC ≥90th percentile. Elevated blood pressure was based on either SBP (>130 mmHg) or DBP (>85 mmHg), high salivary glucose was defined as ≥1.13 mg/dL; equivalent to an extrapolated blood concentration of 100 mg/dL (Mascarenhas et al., 2014) and low salivary HDLC was defined as ≤0.6 mg/dL; equivalent to an extrapolated blood concentration of 40 mg/dL (Singh et al., 2014).

**Table 1:** Characteristics of study subjects based on the scoring system for metabolic syndrome (MetS).

Phenotype	Total population	MetS (score ≥3)	Intermediate (score = 1 or 2)	Non-MetS (score = 0)	*p*-Value^∗^
**Number (n)**	**1313**	**246**	**834**	**233**	
Gender (M/F)	472/841	105/141	293/541	74/159	0.024
Age (years)	12.08 ± 0.64	12.04 ± 0.62	12.08 ± 0.65	12.11 ± 0.61	0.411
Body weight (kg)	53.25 ± 16.70	70.24 ± 15.44	51.24 ± 15.14	42.60 ± 8.39	<0.001
Waist circumference (cm)	80.16 ± 14.56	91.85 ± 14.099	78.84 ± 13.95	72.59 ± 9.14	<0.001
BMI (kg/m^2^)	22.91 ± 5.94	29.26 ± 5.13	22.17 ± 5.35	18.87 ± 2.79	<0.001
Obesity (%)	34.3	91.9	26.8	0	
HDLC in saliva (mg/dL)	0.95 ± 1.50	0.61 ± 1.20	1.01 ± 1.53	2.11 ± 1.95	<0.001
Glucose in saliva (mg/dL)	0.16 ± 0.34	0.26 ± 0.53	0.15 ± 0.31	0.062 ± 0.05	<0.001
Diastolic blood pressure (mmHg)	78.93 ± 13.96	90.57 ± 11.13	78.15 ± 13.45	69.39 ± 8.98	<0.001
Systolic blood pressure (mmHg)	116.37 ± 16.74	131.02 ± 13.35	115.27 ± 15.7	104.87 ± 12.04	<0.001
Fitness (beats/min)^#^	21.33 ± 14.85	25.75 ± 13.69	23.56 ± 13.66	21.84 ± 13.96	0.012
Saliva flow rate (ml/h)	26.40 ± 45.12	27.30 ± 18.67	27.07 ± 51.29	22.96 ± 15.61	0.41
Heart rate (beats/min)	91.14 ± 25.90	93.38 ± 15.25	89.88 ± 13.89	93.26 ± 53.39	0.065
Sleep (hours/week)	8.96 ± 3.10	8.70 ± 3.21	9.00 ± 3.09	9.05 ± 3.05	0.421


*^#^Fitness; increase in heart rate following 3 min of standardized exercise. cm, Centimeter; kg/m^2^, kilogram/meter square; mg/dL, kilogram/meter square, milligram per deciliter; mm Hg, millimeter of mercury. ^∗^p-Value calculated using one-way ANOVA test.*

### Metabolic Syndrome Score

Metabolic syndrome was first recognized as a syndrome delineating the co-occurrence of common metabolic traits such as obesity, insulin resistance, hypertension, impaired glucose tolerance or diabetes, hyperinsulinemia, and dyslipidemia (Reaven, 1988). Although definitions of MetS remain controversial, a method adopted in National Health and Nutrition Examination Survey (NHANES) III, defines MetS as the presence of any *3 of these 5 clinical diagnostic traits* (Ford et al., 2002). These characteristics were adapted in 2007 to 10 to 16-year-old adolescents by a consensus group of the International Diabetes Foundation as (1) abdominal girth (≥90th percentile in waist circumference), (2) high blood pressure (SBP ≥ 130 mmHg or DBP ≥ 85 mmHg), (3) reduction in HDLC (<40 mg/dL), (4) high fasting glucose (≥100 mg/dL), and (5) high fasting triglyceride (≥150 mg/dL) (Zimmet et al., 2007). In our study cohorts, MetS was defined based on the presence of at least three of the following four risk factors, *(1) increased WC (≥90th percentile); (2) raised blood pressure (SBP ≥130 or DBP ≥85; (3) reduced salivary HDLC level (<0.6 mg/dL); and (4) elevated salivary glucose (≥1.13 mg/dL)*. We have previously shown that the salivary measures of HDLC (Shi et al., 2015) and glucose (Hartman et al., 2016) correlates with the plasma measurements. Since salivary triglycerides were not observed to correlate with plasma triglycerides, this was not measured. Following the criterion (Reaven, 1997; Ford et al., 2002; Zimmet et al., 2007; Hartman et al., 2015; Shi et al., 2015), a total of 246 children were classified as MetS (a score ≥3). About 99.1% of children in this group are obese with two or more additional metabolic conditions. A subset of 233 children who had no indications of MetS were considered as non-MetS (score = 0).

A subgroup of 834 children who failed to fulfill the comprehensive definition of MetS were classified as intermediate metabolic/ unclassified group. The children in this group majorly differs from the MetS by the presence of one or two metabolic risk factors (score = 1 or 2) and comprises of 26.8% of obese, 35.5% of SBP/DBP and 61.5% of low HDLC children. Hence, the intermediate risk group was considered as a separate subgroup for subsequent analysis. It is not justified to label this group as MetS (given the strict criteria), nor as non-MetS, as they are characterized by the presence of at least one or two metabolic risk factors and may fulfill the criteria for MetS later in life as they grow older. Given the fact that MetS is age-dependent, we opted for a thorough and more scientifically transparent approach. Both MetS and intermediate MetS groups were separately compared to the group children with no metabolic complications (Non-MetS).

### SNP Genotyping Using Real-Time PCR

Caveolin-1 rs1997623 SNP genotyping was conducted using the TaqMan SNP genotyping assay (Applied Biosystems, Foster City, CA, United States) and the ABI 7500 real-time PCR system (Applied Biosystems). Each PCR reaction contained 10 ng of genomic DNA, 5× FIREPol Master Mix (Solis BioDyne, Tartu, Estonia, Europe) and 1 μl of 20× TaqMan SNP Genotyping Assay. Thermal cycling conditions were as follows: 60°C for 1 min, 95°C for 15 min, 40 cycles of 95°C for 15 s and 60°C for 1 min. The genotypes ascribed by real-time PCR were confirmed by direct sequencing of the PCR products for selected cases of homozygotes and heterozygotes. Sequencing reactions were performed using the BigDye terminator cycle sequencing FS ready reaction kit (Applied Biosystems) according to manufacturer’s instructions on an ABI PRISM 3730 Xl genetic analyzer (Applied Biosystems).

### Statistical Analysis

Genetic association analysis was conducted using the Statistical Package for the Social Sciences, SPSS version 25.0’ (IBM Corp., Armonk, NY, United States). Deviation from the Hardy–Weinberg equilibrium (HWE) was tested using the GenePop software^1^. Differences in genotype and allele frequencies between the study groups were assessed using the chi-square (χ^2^) test *p* ≤ 0.05 was considered statistically significant. Genotype-based ORs and 95% confidence interval (CI) were calculated to measure potential risk factors using individuals homozygous for the non-susceptible allele as a reference. To further analyze the overall effect of rs1997623 variant on studied phenotype individuals carrying the mutant allele (CA and AA) were combined. For continuous variables, comparisons of the means between study subjects were assessed using the Student’s *t*-test or ANOVA for normally distributed data and the non-parametric Mann–Whitney *U*-test for non-normally distributed data. To assess the effect of rs1997623 genotype on continuous variable linear regression analysis was adopted. Binary logistic regression analysis was used to identify the risk of different genotypes of rs1997623 variant for MetS and low measures of HDLC after adjustment for the confounding effect of age and gender. The rs1997623 variant was considered as a predictor variable and binary outcome measures of metabolic status (presence/absence) as a predicted variable. The wildtype CC genotype was used as the reference category to calculate the adjusted odds ratio and 95% CI. Two-way ANOVA was carried out using GraphPad prism software^2^. Differences in the allelic frequency distribution of rs1997623 variant between Kuwaiti and other ethnic population from GnomAD exome population database^3^ were carried out using chi-square test (2 × 2, 1df).

## Results

### Study Characteristics

Genetic profiling of ancestry was not carried out to due to limitations with the salivary DNA samples. Ethnic bias within the population studied was minimized by excluding non-native children from Asia and adjacent Arab countries. This was in turn facilitated by conducting a detailed interview of the study participants to understand their ethnic background and ancestry. DNA samples of native Kuwaiti children who attended public schools where included in this study. A total of 1,313 children were genotyped to study the potential association of the *CAV1* rs1997623 C/A SNP with specific metabolic traits. The number of live births in Kuwait in 2002 was 43,490 (United Nations Demographic Yearbooks). Therefore, the 1,313 children in this study represent approximately 3% of the target population of native Kuwaiti children, who were 10-years old in 2012.

The characteristics of study participants based on their metabolic status are detailed in Table 1. About a third of the children (34.3%), were obese. Two hundred and forty-six children (18.7%) were classified as having MetS; 834 children (63.5%) were in the intermediate metabolic risk group and 233 children (17.7%) had no indication of MetS. Obesity was highly prevalent in the MetS group (91.9%), while 26.8% of children were obese in the intermediate metabolic group. None of the children were obese in the group without MetS. As expected, significantly increased measures of body weight, WC, BMI, HDLC, glucose, DBP, SBP, and fitness were observed in the MetS group followed by intermediate MetS in comparison to non-MetS group, by one-way ANOVA analysis (Table 1). Other tested parameters such as age, saliva flow rate, heart rate, and weekly sleeping hours failed to show any significant differences across the three study groups.

We further observed a significant correlation between various tested phenotypes such as weight, BMI, waist circumference, SBP, DBP and fitness. Both glucose and HDLC failed to show any significant correlation with the aforementioned phenotypes, though a weak positive correlation was observed to exist between glucose and HDLC (*p* < 0.001, *r* = 19.4). The analysis stratified by sex failed to show any significant differences in the distribution of obesity, hypertension, HDLC and glucose between boys and girls. No significant deviation from HWE was found for the tested SNP (*p* > 0.05); allele-A represents the minor allele with a frequency of 0.15 in the tested population. Studying the genotype–phenotype relationships revealed significant differences in the distribution of WC, weight, BMI, DBP, SBP, fitness, glucose levels, and HDLC levels between MetS, intermediate MetS and the group without MetS, as determined using two-way ANOVA (*p* < 0.0003, Supplementary Table 1).

### Association of *CAV1* rs1997623 SNP With MetS

**FIGURE 1:**
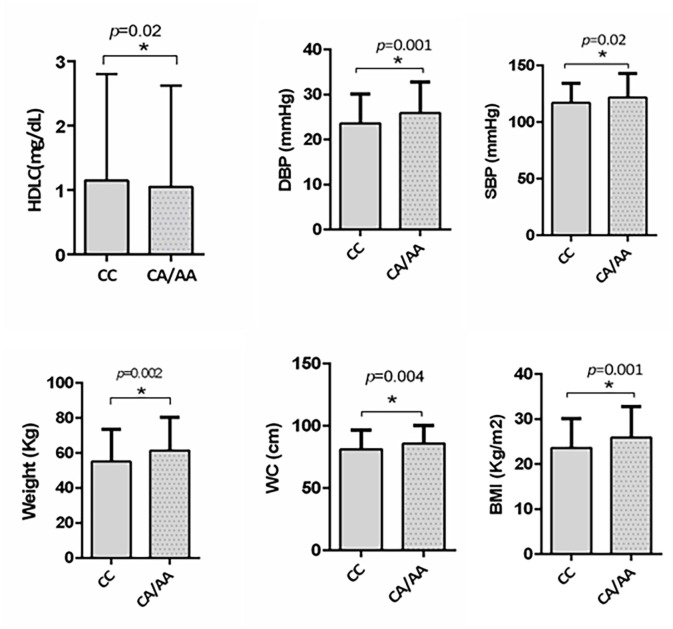
Depicts significantly increased measures of weight (*p* = 0.002), BMI (*p* = 0.001), waist circumference (*p* = 0.004), DBP (*p* = 0.007), and SBP (*p* = 0.02) in subjects carrying mutant CA/AA genotype (*n* = 115) compared to those with wildtype CC genotype (*n* = 358) in a subgroup of subjects with and without MetS by independent *t*-test. A significant reduction in HDLC (*p* = 0.02) was also observed in subjects carrying mutant CA/AA genotype compared to the wildtype CC genotype by Mann–Whitney test.

We further investigated the risk role of rs1997623 variant by linear regression analysis with the MetS score as a continuous dependent variable and genotype as the independent variable (CC, CA/AA) controlling for other covariates such as age and gender. Analysis revealed significant association of rs1997623 variant with MetS (*F* = 4.43, *p* = 0.004). A subgroup of children with and without MetS, excluding the intermediate group, also revealed significantly increased measures of body weight (*p* = 0.002), BMI (*p* = 0.001), WC (*p* = 0.004), DBP (*p* = 0.001), and SBP (*p* = 0.02) in the mutant CA/AA genotypes compared with those carrying the wild-type (CC) genotype (Figure 1).

Categorical analysis revealed a significant difference in the distribution of the rs1997623 variant across the three study groups (χ^2^ = 11.64, *p* = 0.02; df = 4). The rs1997623 variant was observed to be significantly associated with susceptibility to MetS; the frequency of the A-allele was higher in the MetS group (15%) than that in the non-MetS group (10%), with a significant χ^2^ value of 6.24 (*p* = 0.01) and an OR of 1.66 at 95% CI 1.11–2.46 (Table 2). The heterozygous rs1997623 CA genotype showed significant association with MetS compared with the non-MetS group (*p* = 0.005, OR = 1.88 at 95% CI 1.21–2.93). Lack of association between MetS and mutant AA genotype could be due to limited frequency of AA genotype in the study population. We further observed significant associations of the rs1997623 A-allele (*p* = 0.002, OR = 1.71 at 95% CI 1.22–2.40) and CA genotype (*p* = 0.005, OR = 1.70 at 95% CI 1.17–2.47) with the intermediate metabolic group compared with the non-MetS group (Table 2). The overall distribution of genotype frequencies (CA + AA) versus CC) also revealed significant association of the rs1997623 SNP with MetS (*p* = 0.01, OR = 1.75 at 95% CI 1.13–2.68) and with the intermediate metabolic group (*p* = 0.004, OR = 1.7 at 95% CI 1.18–2.44).

**Table 2:** Allele and genotypic distribution of the *CAV1* rs1997623 SNP based on metabolic syndrome risk score.

rs1997623/Allele	MeS (score = 3 or 4)	Intermediate group (score = 1 or 2)	Non-MeS (score = 0)
Allele C	413 (0.85)	1391 (0.85)	412 (0.90)
Allele A	73 (0.15)	255 (0.15)	44 (0.10)
*p*-value^∗^	0.01	0.002	
OR (95% CI)	1.66 (1.11–2.46)	1.71 (1.22–2.40)	

**rs1997623/Genotype**	**MeS (score = 3 or 4)**	**Intermediate group (score = 1 or 2)**	**Non-MeS (score = 0)**

Homozygous wild type CC	172 (0.71)	587 (0.71)	186 (0.81)
*p*-value^∗^	0.01	0.004	
OR (95% CI)	0.57 (0.37–0.88)	0.59 (0.40–0.84)	
Heterozygous CA	69 (0.28)	217 (0.26)	40 (0.17)
*p*-value^∗^	0.005	0.005	
OR (95% CI)	1.88 (1.21–2.93)	1.70 (1.17–2.47)	
Homozygous mutant AA	2 (0.01)	19 (0.02)	4 (0.02)
*p*-value^∗^	0.44	0.600	
OR (95% CI)	0.45 (0.085–2.59)	1.34 (0.45–3.96)	
Compiled (CA+AA) vs. CC	71 (0.29)/172 (0.71)	236 (0.29)/587 (0.71)	44 (0.19)/186 (0.81)
*p*-value^∗^	0.01	0.004	
OR (95% CI)	1.75 (1.13–2.68)	1.7 (1.18–2.44)	


*^∗^p-value was calculated using Chi-square test. Overall distribution of rs1997623 across three study groups also showed significant association with χ^2^ = 11.64, p = 0.02 (df4).*

We additionally carried out logistic regression analysis to ascertain the effects of rs1997623, age and gender on the like-hood of children having MetS. Our analysis indicates that only gender contributed significantly to model with a *p* = 0.006. Compared to wildtype CC genotype, children with CA genotype showed increased susceptibility to MetS with a *p* = 0.004 and adjusted odds ratio (AOR) of 1.938 at 95% CI 1.24–3.029 (Table 3). To further analyze the overall effect of rs1997623 variant on the tested phenotype, we combined children carrying the mutant allele (CA and AA). Compared to wildtype CC, children carrying the mutant genotype showed increased susceptibility to MetS with a *p* = 0.008 and adjusted odds ratio (AOR) of 1.806 at 95% CI 1.170–2.789.

**Table 3:** Logistic regression analysis of the rs1997623 variant in a group of subjects with and without metabolic syndrome.

With MetS (score = 3 or 4)/Without MetS (score = 0)	*p*-value^∗^	AOR	95% CI
Sex			
*Male (Reference)*			
*Female*	0.006	0.582	0.397–0.853
Age	0.193	0.819	0.606–1.106
RS1997623			
*CC (Reference)*			
*CA*	0.004	1.938	1.240–3.029
*AA*	0.467	0.527	0.094–2.967
RS1997623			
*CC (Reference)*			
*CA + AA*	0.008	1.806	1.170–2.789


*^∗^p-value was adjusted for age and gender. AOR, adjusted odds ratio.*

### Association of *CAV1* rs1997623 SNP With Low Salivary High-Density Lipoprotein Cholesterol (HDLC)

**FIGURE 2:**
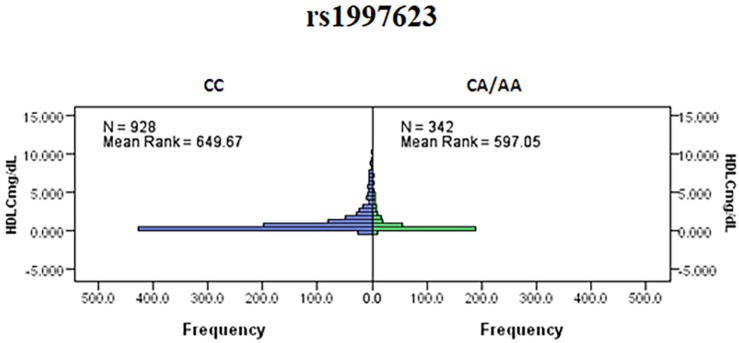
Indicates a significant association of rs1997623 with low HDLC in Kuwaiti children. Subjects carrying the heterozygous or mutant genotype showed significantly lower level of HDLC when compared to those with the wildtype genotype by Mann–Whitney *U*-test (*p* = 0.023).

We further investigated the association of the rs1997623 SNP with metabolic traits such as saliva HDLC of the study participants. Our analysis revealed a significant association of rs1997623 with low saliva HDLC in Kuwaiti children. Subjects carrying the heterozygous or mutant genotype CA/AA (mean rank 597.05, *n* = 342) had a significantly lower HDLC level compared with those carrying the CC wildtype (mean rank 649.67, *n* = 928), (*p* = 0.023; Figure 2). The linear regression analysis failed to show a statistically significant association between HDLC and rs1997623 SNP.

We further verified the association of the rs1997623 SNP with low HDLC (Table 4) by stratifying the participants into clinical subtypes as follows: those with a low HDLC level (*n* = 724) and those with a normal HDLC level (*n* = 572). The frequency of the A-allele was higher in subjects with low HDLC (16%) than those in the control group (13%), χ^2^ = 6.08; (*p* = 0.01) and an OR = 1.33 (95% CI 1.05–1.66). Similarly, the rs1997623 heterozygous CA genotype showed significant association with predisposition toward low HDLC (*p* = 0.0008; OR = 1.55; 95% CI 1.20–2.01). The overall distribution of genotype frequencies (CA + AA)/CC, also showed significant association (*p* = 0.003, OR = 1.47, 95% CI 1.14–1.89) with low HDLC. The strength of this association significantly improved after adjustment for age and sex using logistic regression analysis (Table 5). The children carrying CA genotype showed increased odds of low HDLC level compared to those carrying CC genotype (*p* = 0.001, AOR = 1.569, 95% CI 1.209–2.1037). Combined analysis also indicated association of mutant genotype with reduced measured of HDLC (*p* = 0.002, AOR = 1.489, 95% CI 1.157–1.916).

**Table 4:** Allele and genotypic distribution of the *CAV1* rs1997623 SNP in low HDLC subjects compared to the control group.

rs1997623	LHDLC	NHDLC	*p*-value	OR	95% CI
C	1216 (0.84)	1000 (87)	0.01	1.33	1.05–1.658
A	232 (0.16)	144 (0.13)			
	
	**LHDL (724)**	**NHDLC (572)**	***p*-value**	**OR**	**95% CI**
	
CC	504 (0.70)	441 (0.77)	0.002	0.68	0.53–0.88
CA	208 (0.29)	118 (0.21)	0.0008	1.55	1.197–2.008
AA	12 (0.02)	13 (0.02)	0.42	0.725	0.33–1.601


*CI, confidence interval; LHDLC, low high-density lipoprotein cholesterol; NHDLC, normal high-density lipoprotein cholesterol; OR, odds ratio.*

**Table 5:** Logistic regression analysis of the rs1997623 variant in a group of subjects with low HDLC and normal HDLC.

LHDLC/NHDLC	*p*-value^∗^	AOR	95% CI
Sex			
*Male (Reference)*			
*Female*	0.017	0.754	0.598–0.950
Age	0.055	0.844	0.709–1.004
RS1997623_CV2			
*CC (Reference)*			
*CA*	0.001	1.569	1.209–2.1037
*AA*	0.538	0.778	0.350–1.729
RS1997623_CV2			
*CC (Reference)*			
*CA + AA*	0.002	1.489	1.157–1.916


*^∗^p-value was adjusted for age and gender. AOR, adjusted odds ratio; CI, confidence interval; LHDLC, low high-density lipoprotein cholesterol; NHDLC, normal high-density lipoprotein cholesterol.*

### Distribution of the rs1997623 Variant in the Kuwaiti Population Compared With Other Ethnicities

**FIGURE 3:**
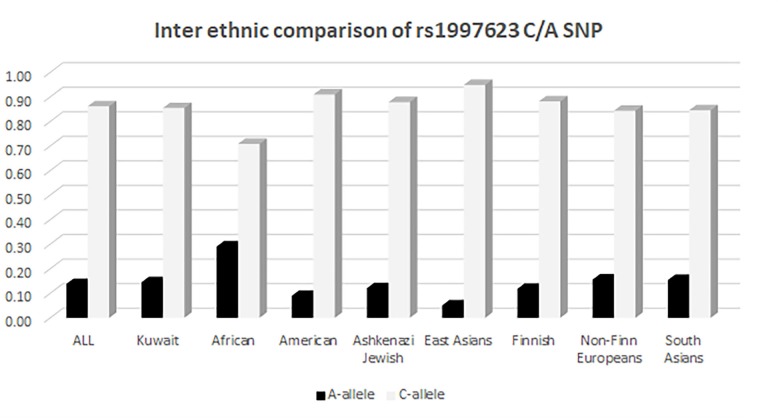
Inter ethnic comparison of rs1997623 C/A SNP in Kuwaitis with its distribution in GnomAD exome population database (http://gnomad.broadinstitute.org).

We further compared the distribution of the rs1997623 variant in Kuwaitis with its distribution in the GnomAD exome population database (see text footnote 3). The comparison of allele frequency (Figure 3) was carried out using a χ^2^ (2 × 2, 1df). The minor allele frequency of rs1997623 was observed to be 0.15 in the Kuwaiti population. The rs1997623 A-allele was found to be the minor allele in all the tested populations. African population showed the highest frequency of the A-allele (0.29), while the East Asians were reported to have the lowest frequency (0.05). A comparative analysis indicated a highly significant difference in allele frequency distribution between Kuwaitis and Africans (*p* < 0.0001), Americans (*p* < 0.0001), Ashkenazi Jews (*p* = 0.001), East Asians (*p* < 0.0001), and Finns (*p* < 0.0001). However, non-Finn Europeans (*p* = 0.123) and South Asians (*p* = 0.225) failed to show any significant differences when compared with Kuwaitis. Similarly, the Avon Longitudinal Study of Parents and Children (ALSPAC) (*p* = 0.862) and TwinsUK cohorts (*p* = 0.152) revealed no significant differences when compared with the Kuwaiti population.

## Discussion

Our exome variant database hinted toward a possible association between the rs1997623 and obesity. However, the data was too limited to reach any meaningful conclusion, which prompted us to undertake a more extensive study based on 1313 children from all Governorates of Kuwait. In this study, saliva samples were used as specimens to analyze the association of the *CAV1* rs1997623 SNP with metabolic complications related to obesity.

Variations in the *CAV1* gene have been recently associated with metabolic complications including cardiovascular outcomes (Pojoga et al., 2011; Baudrand et al., 2015; Chen et al., 2016). To our knowledge no study has so far characterized the role of *CAV1* rs1997623 variant with MetS. In the presented study, we report a novel association of *CAV1* rs1997623 variant with the MetS and with low saliva HDLC levels in Kuwaiti children. The heterozygous rs1997623 variant leads to significantly lower levels of HDLC in the study population. HDLC is considered as the “good cholesterol,” that act as a scavenger mediating the transport of low density lipoprotein from the arteries to the liver. Apart from its anti-oxidant, anti-thrombotic and anti-inflammatory effects, HDLC tends to promote pancreatic beta cell function and glucose metabolism (Drew et al., 2009; Fryirs et al., 2010). The lack of association of homozygous mutant AA genotype with decreased odds of MetS could be due to limited frequency of AA genotype in the study population. Further analysis need to be carried out in a relatively large cohort to estimate the effect of mutant genotype. Mitigating our selection, we additionally detected a significant association of *CAV1* rs1997623 variant with intermediate metabolic group. This possibly reflects the association of rs1997623 variant with individual predictive markers of MetS, further signifying the need to address the children with fewer metabolic complications.

In contrast to candidate gene studies, none of the GWA studies implicated in the literature have detected the significance of *CAV1* variants with respect to metabolic traits. To the best of our knowledge, the most commonly used Illumina Human OmniExpress Bead Chip genome wide array does not include the tested rs1997623 variant. Apparently, a vast majority of GWA studies were also performed in European, African-American and Asian population and relatively little information is available from the State of Kuwait (Hebbar et al., 2017, 2018). The genetic divergence of Kuwaiti population has largely been evidenced by the differences in disease prevalence and risk allele frequencies. The regions socio economic status post-hydrocarbon boom and sedentary lifestyle practices have attributed to the increase in prevalence of metabolic disorders including dyslipidemia (70.3%), obesity (48.2%), hypertension (25.3%), and diabetes (17.9%) (Alarouj et al., 2013). Although differences are small, a comparative analysis of allelic frequency distribution of rs1997623 variant in Kuwaiti population, at this locus, is significantly different from African, American, Finnish, Jewish, and East Asian populations, while being closer to South Asian and non-Finnish European populations.

Not much is known regarding the functional consequences of the rs1997623 SNP. The rs1997623 varint is part of the exon 1 transcript (NM_001172895.1) that undergoes nonsense-mediated decay. We are currently performing functional studies on this variant in our laboratory, specifically addressing whether the variant alters the expression of caveolin *in vitro*. Several animal and cell model studies have elucidated the role of *CAV1* in lipid metabolism. *CAV1* knockout mice were reported to have hyper-triglyceridaemia with adipocyte abnormalities (Razani et al., 2002). Parallel to this, significantly decreased expression of the *CAV1* gene positively correlates with reduced lipogenic gene expression in the visceral adipose tissue of obese subjects (Fernandez-Real et al., 2010). The role of *CAV1* in HDLC metabolism was further evidenced by increased levels of plasma HDLC in mouse liver overexpressing *CAV1* (Frank et al., 2001). Given the fact that the rs1997623 variant is predicted to be associated with transcript-dependent promoter loss, we hypothesize that the magnitude of effect caused by the rs1997623 SNP would largely rely on transcript-dependent expression of *CAV1*. In addition to the allelic variations of the rs1997623 SNP, the variable ratio of the various *CAV1* isoforms may also be important in understanding its regulatory role in lipid metabolism.

## Conclusion

We investigated the genetic associations of the rs1997623 variant for its potential association with metabolic traits in a large cohort of Kuwaiti children. Our study revealed, for the first time, an association of the rs1997623 variant with MetS in Kuwaiti children with high prevalence of obesity. Heterozygosity of the rs1997623 variant was associated with lower levels of HDLC in this study group. This study calls for further in-depth population-based studies to unravel the genetics of obesity and related metabolic complications in the Kuwaiti/Arab populations.

## Data Availability Statement

The raw data supporting the conclusions of this manuscript will be made available by the authors, without undue reservation, to any qualified researcher.

## Author Contributions

RN and FA-M designed the study. FA-M, RN, and JG directed the work. OA, JG, SD, and M-LH directed study participant recruitment, sample processing, and data collection. FA-M, RN, HaA, and MB shortlisted the gene/variant. RN, MM, GA, and NA performed the experiment. FA-M, RN, and PS carried out statistical analysis and interpretation. EA-O, LD, HeA, M-LH, MT, HoA, and FA-R carried out clinical data analysis and interpretation. RN and FA-M wrote the manuscript. EA-O, JT, LD, JG, AA, FA-R, SS, MQ, MA-F, JA, M-LH, PS, MB, HaA, and OA reviewed and edited the manuscript. FA-M, EA-O, and JT critically revised and approved the manuscript.

## Conflict of Interest Statement

The authors declare that the research was conducted in the absence of any commercial or financial relationships that could be construed as a potential conflict of interest.

## Footnotes

1 http://genepop.curtin.edu.au

2 https://www.graphpad.com/scientific-software/prism

3 http://gnomad.broadinstitute.org
