# Corrigendum: Caveolin-1 Variant Is Associated With the Metabolic Syndrome in Kuwaiti Children

**DOI:** 10.3389/fgene.2019.00221

**Published:** 2019-03-20

**Authors:** Rasheeba Nizam, Ebaa Al-Ozairi, Jo Max Goodson, Motesam Melhem, Lena Davidsson, Hessa Alkhandari, Ashraf Al Madhoun, Sara Shamsah, Malak Qaddoumi, Ghazi Alghanim, Nouf Alhasawi, Mohamed Abu-Farha, Jehad Abubaker, Ping Shi, Mor-Li Hartman, Mary Tavares, Milad Bitar, Hamad Ali, Hossein Arefanian, Sriraman Devarajan, Faisal Al-Refaei, Osama Alsmadi, Jaakko Tuomilehto, Fahd Al-Mulla

**Affiliations:** ^1^Functional Genomics Unit, Dasman Diabetes Institute, Kuwait City, Kuwait; ^2^Clinical Division, Dasman Diabetes Institute, Kuwait City, Kuwait; ^3^Applied Oral Sciences, The Forsyth Institute, Cambridge, MA, United States; ^4^Family Medicine and Pediatric Unit, Dasman Diabetes Institute, Kuwait City, Kuwait; ^5^Faculty of Allied Health Sciences, Kuwait University, Kuwait City, Kuwait; ^6^Biochemistry and Molecular Biology Unit, Dasman Diabetes Institute, Kuwait City, Kuwait; ^7^Faculty of Medicine, Kuwait University, Kuwait City, Kuwait; ^8^Immunology Unit, Dasman Diabetes Institute, Kuwait City, Kuwait; ^9^National Dasman Diabetes Biobank, Dasman Diabetes Institute, Kuwait City, Kuwait; ^10^Cell Therapy and Applied Genomics, King Hussein Cancer Center, Amman, Jordan; ^11^Research Division, Dasman Diabetes Institute, Kuwait City, Kuwait

**Keywords:** *CAV1*, HDLC, metabolic syndrome, Kuwaiti children, obesity

Author names were incorrectly spelled as “Mark L. Hartman”, “Jihad Abubaker”, “Ebaa Alozairi”, and “Mohamed Abufarha”. The correct spelling is “Mor-Li Hartman”, “Jehad Abubaker”, “Ebaa Al-Ozairi”, and “Mohamed Abu-Farha”.

The updated author contributions statement appears below.

“RN and FA-M designed the study. FA-M, RN, and JG directed the work. OA, JG, SD, and M-LH directed study participant recruitment, sample processing, and data collection. FA-M, RN, HaA, and MB shortlisted the gene/variant. RN, MM, GA, and NA performed the experiment. FA-M, RN, and PS carried out statistical analysis and interpretation. EA-O, LD, HeA, M-LH, MT, HoA, and FA-R carried out clinical data analysis and interpretation. RN and FA-M wrote the manuscript. EA-O, JT, LD, JG, AA, FA-R, SS, MQ, MA-F, JA, M-LH, PS, MB, HaA, and OA reviewed and edited the manuscript. FA-M, EA-O, and JT critically revised and approved the manuscript.”

The authors apologize for this error and state that this does not change the scientific conclusions of the article in any way. The original article has been updated.

