# Supplementary material for: Caveolin-1 Variant Is Associated With the Metabolic Syndrome in Kuwaiti Children
Source: Front Genet. 2018 Dec 21;9:689. doi: 10.3389/fgene.2018.00689 (PMC6308323; doi:10.3389/fgene.2018.00689)
Supplement: Supplementary file 1 [file Table_1.doc]

Supplementary Material

**Caveolin-1 variant is associated with the metabolic syndrome in Kuwaiti children**

Rasheeba Nizam^1^*, Ebaa Alozairi^2^, Jo Max Goodson^3^, Motesam Melhem^1^, Lena Davidsson^4^, Hessa Alkhandari^4^, Ashraf Al Madhoun^1^, Sara Shamsah^5^, Malak Qaddoumi^1^, Ghazi Alghanim^1^, Nouf Alhasawi^1^, Mohamed Abufarha^6^, Jihad Abubaker^6^, Ping Shi^3^, Mark L. Hartman^3^, Mary Tavares^3^, Milad Bitar^7^, Hamad Ali^5^, Hossein Arefanian^8^, Sriraman Devarajan^9^, Faisal Al-Refaei^2^, Osama Alsmadi^10^, Jaakko Tuomilehto^11^ and Fahd Al-Mulla^1^*

*** Correspondence:** Fahd Al-Mulla: [fahd.almulla@dasmaninstitute.org/](mailto:fahd.almulla@dasmaninstitute.org/) Rasheeba Nizam: rasheeba.iqbal@dasmaninstitute.org

**Supplementary table 1:** Distribution of study phenotypes based on rs1997623 genotypes in study subjects based on the scoring system for metabolic syndrome (MetS)

| **Trait** | **Genotypes** | **MetS (Score ≥3)** | | **Intermediate (Score 1-2)** | | **Non MetS (Score =0)** | | | **F (DFn, DFd)** | **p-value** |
| --- | --- | --- | --- | --- | --- | --- | --- | --- | --- | --- |
|  |  | **Mean ± SD** | **N** | **Mean ± SD** | **N** | **Mean ± SD** | **N** |  | |  |
| WC | CC | 90.97 ± 15.14 | 172 | 79.32 ± 13.99 | 587 | 72.23 ± 8.95 | 186 | F (2, 1290) = 126.3 | | *p*<0.0001 |
|  | CA/ AA | 93.62 ± 11.19 | 71 | 77.62 ± 13.96 | 236 | 73.48 ± 9.84 | 44 |  |  |  |
| Weight | CC | 69.27 ± 15.54 | 172 | 51.37 ± 15.44 | 586 | 42.13 ± 8.31 | 186 | F (2, 1289) = 220.1 | | *p*<0.0001 |
|  | CA/ AA | 72.21 ± 15.33 | 71 | 50.94 ± 14.54 | 236 | 44.14 ± 8.30 | 44 |  |  |  |
| BMI | CC | 28.87 ± 5.11 | 172 | 22.26 ± 5.57 | 586 | 18.27 ± 2.73 | 186 | F (2, 1289) = 260.9 | | *p*<0.0001 |
|  | CA/ AA | 30.07 ± 5.14 | 71 | 21.94 ± 4.83 | 236 | 19.26 ± 2.91 | 44 |  |  |  |
| DBP | CC | 89.95 ± 11.11 | 172 | 78.23 ± 13.22 | 587 | 69.27 ± 9.01 | 185 | F (2, 1289) = 173.6 | | *p*<0.0001 |
|  | CA/ AA | 92.21 ± 11.24 | 71 | 77.72 ± 14.11 | 236 | 69.45 ± 8.93 | 44 |  |  |  |
| SBP | CC | 130.17 ± 12.85 | 172 | 115.28 ± 15.60 | 587 | 105.13 ± 10.67 | 186 | F (2, 1290) = 197.4 | | *p*<0.0001 |
|  | CA/ AA | 133.23 ± 14.28 | 71 | 115.16 ± 16.18 | 236 | 103.13 ± 16.72 | 44 |  |  |  |
| HR | CC | 94.18 ± 15.51 | 172 | 90.14 ± 13.61 | 587 | 94.32 ± 59.54 | 185 | F (2, 1289) = 0.8604 | | *p*=0.4232 |
|  | CA/ AA | 91.28 ± 14.69 | 71 | 89.07 ± 14.70 | 236 | 89.48 ± 10.64 | 44 |  |  |  |
| SFR | CC | 27.07 ± 19.07 | 161 | 27.96 ± 60.0 | 545 | 23.48 ± 16.53 | 176 | F (2, 1197) = 0.8865 | | *p*=0.4124 |
|  | CA/ AA | 28.04 ± 17.89 | 66 | 24.72 ± 17.09 | 215 | 20.99 ± 11.29 | 40 |  |  |  |
| Fitness | CC | 25.15 ± 13.58 | 154 | 24.06 ± 13.99 | 517 | 22.35 ± 14.51 | 167 | F (2, 1161) = 8.056 | | *p*=0.0003 |
|  | CA/ AA | 27.32 ± 14.09 | 69 | 22.46 ± 12.77 | 219 | 19.33 ± 11.11 | 41 |  |  |  |
| Glucose | CC | 0.30 ± 0.58 | 172 | 0.14 ± 0.30 | 583 | 0.06 ± 0.05 | 182 | F (2, 1276) = 15.23 | | *p*<0.0001 |
|  | CA/ AA | 0.18 ± 0.38 | 70 | 0.17 ± 0.35 | 231 | 0.06 ± 0.06 | 44 |  |  |  |
| HDLC | CC | 0.64 ± 1.22 | 172 | 1.02 ± 1.56 | 579 | 2.05 ±1.95 | 177 | F (2, 1264) = 66.24 | | *p*<0.0001 |
|  | CA/ AA | 0.51 ± 1.19 | 70 | 0.96 ± 1.45 | 230 | 2.42 ± 2.00 | 42 |  |  |  |

*p*-value was calculated using two-way ANOVA, across the MetS, intermediate and Non-MetS groups. WC-waist circumference, BMI-basal metabolic rate, DBP-Diastolic pressure, SBP-systolic measure, HR- Heart rate, SFR-Saliva Flow rate, HDCL- High density lipoprotein.
